# A New Method for Fitting Bands and Clasps to Teeth

**Published:** 1872-06

**Authors:** A. H. Fuller


### ARTICLE VII.

### *A New Method for Fitting Bands and Clasps to Teeth.*

BY A. H. FULLER, D.D.S.

In "regulating cases," having at times found it extremely difficult to *perfectly* fit a band to a tooth, which I desired to twist, it appeared to me that the difficulty could be overcome in the manner which I will describe, and which I have since found of great advantage, saving time and patience, and giving a more accurate adaptation than could be obtained by any other method with which I am acquainted.

I take an impression of the tooth, from which I get a plaster model; around this I wind a fine platinum wire, one coil above the other, until a band of sufficient width is formed. Over this, and while yet on the plaster model, flow gold solder, and you will have a perfect fitting band. Attach your catches at whatever points on the band you wish to apply your force, by soldering on a small piece of plate, or in any manner the case may suggest to your mind as the best.

Clasps can be perfectly fitted to teeth in this manner, using heavier wire and twenty *karet* gold for solder, cutting the band so as to clasp the tooth in the proper manner.—*Missouri Dental Journal.*

